# Dynamism, Sensitivity, and Consequences of Mesenchymal and Stem-Like Phenotype of Cancer Cells

**DOI:** 10.1155/2018/4516454

**Published:** 2018-10-10

**Authors:** Petra Gener, Joaquin Seras-Franzoso, Patricia González Callejo, Fernanda Andrade, Diana Rafael, Francesc Martínez, Sara Montero, Diego Arango, Joan Sayós, Ibane Abasolo, Simó Schwartz

**Affiliations:** ^1^Drug Delivery and Targeting Group, CIBBIM-Nanomedicine, Vall d'Hebron Institut de Recerca (VHIR), Vall d'Hebron Barcelona Hospital Campus, 08035 Barcelona, Spain; ^2^Networking Research Center on Bioengineering, Biomaterials and Nanomedicine (CIBER-BBN), 08035 Barcelona, Spain; ^3^I3S-Instituto de Investigação e Inovação em Saúde, Universidade do Porto, Porto, Portugal; ^4^INEB-Instituto de Engenharia Biomédica, Universidade do Porto, Porto, Portugal; ^5^Biomedical Research in Digestive Tract Tumors, CIBBIM-Nanomedicine, Vall d'Hebron Institut de Recerca, Universitat Autonoma de Barcelona, Barcelona, Spain; ^6^Immune Regulation and Immunotherapy, CIBBIM-Nanomedicine, Vall d'Hebron Institut de Recerca, Universitat Autonoma de Barcelona, Barcelona, Spain; ^7^Functional Validation and Preclinical Research (FVPR), CIBBIM-Nanomedicine, Vall d'Hebron Institut de Recerca, Universitat Autonoma de Barcelona, Barcelona, Spain

## Abstract

There are remarkable similarities in the description of cancer stem cells (CSCs) and cancer cells with mesenchymal phenotype. Both cell types are highly tumorigenic, resistant against common anticancer treatment, and thought to cause metastatic growth. Moreover, cancer cells are able to switch between CSC and non-CSC phenotypes and vice versa, to ensure the necessary balance within the tumor. Likewise, cancer cells can switch between epithelial and mesenchymal phenotypes via well-described transition (EMT/MET) that is thought to be crucial for tumor propagation. In this review, we discuss whether, and to which extend, the CSCs and mesenchymal cancer cells are overlapping phenomena in terms of mechanisms, origin, and implication for cancer treatment. As well, we describe the dynamism of both phenotypes and involvement of the tumor microenvironment in CSC reversion and in EMT.

## 1. Differences and Similarities of Mesenchymal and Stem-Like Phenotypes of Cancer Cells

Our understanding of cancer biology and genetics has changed sustainably over the past 10 years. We consider tumor to be a highly complex heterogenic dynamic entity that evolves in time, always trying to adapt and survive to adverse conditions. For example, in order to survive to multimodal therapy, which includes resection, chemotherapy, and radiation, tumor cells undergo dynamic clonal evolution. As a result, tumors become a mass of highly heterogeneous cell populations undergoing constant dynamic phenotypic changes [1]. In addition, somatic mutations and phenotypic variations might generate cancer cell clones that develop resistance to treatment and remain progressing while current treatment eliminates only sensitive clones. In fact, a tumor may initially shrink after multimodal treatment, while remaining resistant clones which will survive and eventually cause tumor regrowth and relapse, often rising very aggressive tumor types with unfortunately very limited treatment alternatives [2, 3].

Notably, tumors from patients with recurrent resistant tumors show higher numbers of CSCs and cells with epithelial-mesenchymal transition (EMT) phenotype. Indeed, poor survival has been associated with the presence of both cell types in various clinical trials [4].

**Figure 1:**
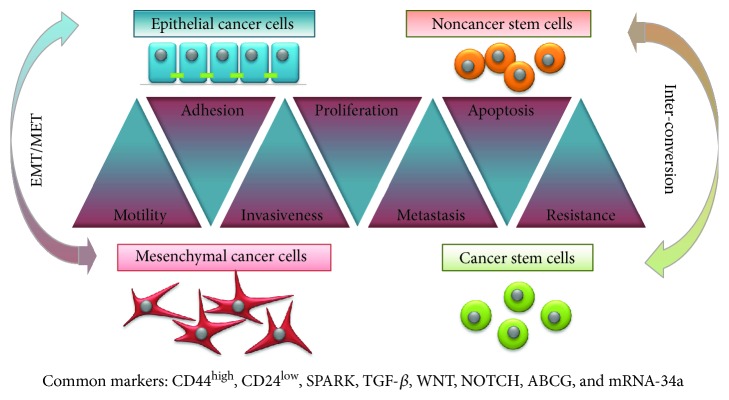
Cancer stem cells versus mesenchymal cancer cells. There are remarkable similarities in the properties of CSCs and cancer cells with mesenchymal phenotype, which oppose from characteristics of non-CSCs and epithelial cancer cells, respectively. Both are highly invasive, tumorigenic, resistant against common anticancer treatment, and thought to cause metastatic growth. Both cell types share several cell markers. Besides, both phenotypes are reversible and can be interchanged via EMT or CSC phenotype interconversion.

CSCs represent a fraction of undifferentiated cancer cells that exhibit stem cell-like features. They have the ability to differentiate and to self-renew. Owing to the phenotypic differences with the rest of tumoral cells, CSCs account for therapy resistance and represent the cellular reserve responsible for tumor regrowth and metastatic spread [5]. CSCs overexpress ATP-dependent drug efflux transporters like P-glycoprotein (P-gp), the multidrug resistance-associated proteins (MRP), and ATP-binding cassette (ABC) transporters at the cell surface, which decrease intracellular drug accumulation. Besides, detoxifying enzymes like aldehyde dehydrogenase 1 (ALDH1A1) and bleomycin hydrolase (BLMH) provide CSCs with further protection against chemotherapy. CSCs are able to enter to a stable quiescence state in hypoxic conditions, overpass the stress condition, and proliferate afterwards [5]. In the last years, many research groups employed big efforts in order to identify biomarkers which could specifically characterize the different subpopulations of CSCs within a tumor [6]. Interestingly, most of the identified CSC markers can be also found in cells with mesenchymal phenotype (CD44^+^/CD24^−^, SPARK, WNT, NOTCH, ABCG, mRNA-34a, etc.). Moreover, the characterization of cancer cells, which have acquired mesenchymal features by EMT, is quite similar to the description of CSCs (Figure 1). EMT cells are essential for tumor progression, including tumor metastasis, therapy resistance, and disease recurrence. A majority of tumors (90%) are epithelial in nature (carcinomas); therefore, the activation of an EMT program, which originally plays a crucial role in organogenesis during embryonic development as well as wound healing and tissue regeneration, can transform epithelial cancer cells into a more aggressive mesenchymal phenotype, promoting local invasion and dissemination at distant organs [7].

**Figure 2:**
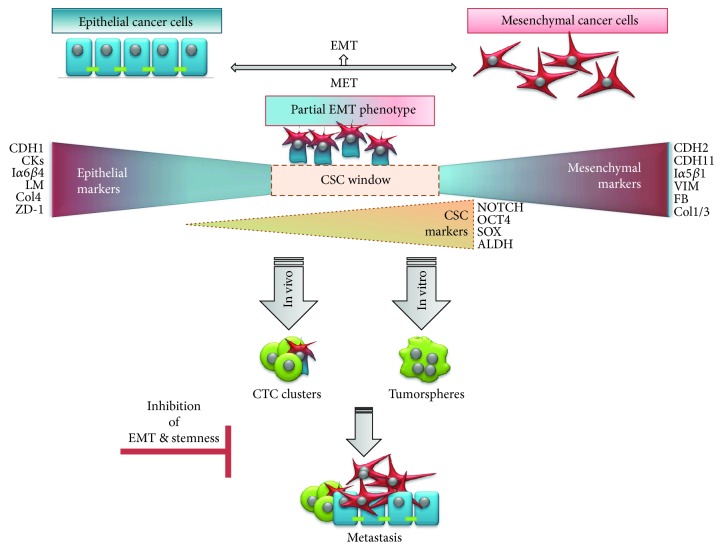
E/M hybrid phenotype. E/M hybrid phenotype of tumor cells represents an ideal window for *stemness* reversion. In this state, cancer cells coexpress epithelial and mesenchymal genes and promote expression of *stemness* genes. This results in formation of a tumor sphere *in vitro* and metastatic spread *in vivo*. Also, a majority of circulating cancer cell (CTC) clusters coexpress epithelial and mesenchymal markers together with stem cell markers. An inhibition of EMT and/or *stemness* phenotype should lead to hindrance of advanced cancer.

During EMT, epithelial cells lose their cell-cell adhesion and apical-basal polarity, gaining the ability to individually migrate and invade basement membrane and blood vessels [7]. This conversion correlates with a decrease in epithelial markers (E-cadherin, cytokeratin, integrin *α*6*β*4, laminins, collagen type IV, ZD-1, etc.) and an increase in mesenchymal markers (N-cadherin, vimentin, fibronectin, cadherin-11, integrin *α*5*β*1, collagen types I and III, etc.) [8–11]. Interestingly, several recent studies pointed out an increase in CSC signature during EMT processes in many carcinomas such as pancreatic, hepatocellular, and colorectal as well as in human mammary epithelial cells [12–15] (Figure 2).

Even though in the past CSCs and EMT were studied independently, accumulating evidence suggests strong parallelisms between EMT activation and CSC formation. EMT is relevant to the acquisition and maintenance of stem cell-like characteristics and is sufficient to endow differentiated normal and cancer cells with stem cell properties. Recently, proteasome activator subunit 3 (PSME3) has been shown to induce epithelial-mesenchymal transition of breast cancer cells together with induction of CSC marker expression and further to influence the tumor immune microenvironment [16]. Moreover, CSCs often exhibit mesenchymal properties within epithelial tumor cells [6, 7, 15, 17–20]. Most likely, heterogeneous cancer cell subpopulations, including CSCs and cells with activated EMT signaling, function in a complementary manner at the collective level to achieve therapeutic resistance and ensure disease progression. The idea of tumors as a highly dynamic heterogeneous mass of cells with an unstable and reversible hierarchy, which seems to be influenced by the origin and biological context of each tumor, is gaining acceptance. According to this scenario, a new concept of tumor plasticity, an “EMT score,” has been proposed to represent the EMT-grade characteristic of each cell line and primary tumor [14, 21]. Tan and colleagues established a universal and quantitative EMT scoring to define an EMT spectrum across various cancers (ovarian, breast, bladder, lung, colorectal, and gastric cancers) [14, 21]. Tumor-specific gene expression was used to establish an EMT scoring method and quantitatively estimated the degree of EMT (−1.0 to +1.0) in a large collection of cell lines and tumors, reflecting epithelial and mesenchymal states as well as the intermediate states that occur during transition. Good correlation between EMT and poorer disease-free survival was observed in ovarian and colorectal cancers, but not in breast cancer or carcinomas. Importantly, a distinct response between epithelial and mesenchymal-like ovarian cancers to therapeutic regimes administered with or without paclitaxel *in vivo* was also observed [14, 21].

Of note, the observed intermediate, mixed epithelial and mesenchymal phenotype (E/M hybrid phenotype), is thought to represent the ideal window for *stemness* reversion [18, 22, 23] (Figure 2). This theory is supported by the fact that repression of EMT is required for effective tumor initiation [24–27] and that CSC reprogramming often involves mesenchymal to epithelial transition (MET) [28, 29].

Further, coexpression of epithelial and mesenchymal genes promotes mammosphere formation and expression of *stemness* genes [22] and drives tumor growth *in vivo* [18, 23]. Besides, according to mathematical models of *stemness*-decision circuits, it has been suggested that a hybrid E/M state is more likely to gain *stemness* than complete EMT is [14, 30]. These observations are consistent with experiments showing that a majority of circulating cancer cells (CTCs) coexpress epithelial and mesenchymal markers together with stem cell markers [31]. CTCs in a semimesenchymal phenotype have higher proliferative and invasive abilities than cells with complete EMT phenotype and are able to originate distant metastasis [32, 33] (Figure 2). The association of a hybrid E/M phenotype with *stemness* is not specific to tumor progression but has been also reported in physiological conditions in adult hepatic stem/progenitor cells (HSCs) and adult renal progenitors upon tissue injury and show to mediate tissue repair and regeneration [34–36].

## 2. Dynamic Cancer Cell Phenotype

There is increasing evidence showing that some cell subpopulations are subjected to a dynamic phenotype within a tumor. Although the importance of the differentiation state of tumor cells on their malignant capacity has been reported since the 80s, the study of the underlying mechanism controlling these cellular states has been neglected until recently [37]. Currently, the most studied phenomenon of cellular differentiation/dedifferentiation processes undergone by tumor cells with influence in cancer progression is transition from epithelial to mesenchymal phenotype and their counter pathway mesenchymal to epithelial transition. Both phenomena have been reported in several cancer types including colorectal cancer, breast cancer, prostate cancer, pancreatic cancer, bladder cancer, and lung cancer, among others. EMT cellular conversion has been extensively studied during the last decade. In this regard, three main molecular pathways leading and regulating this process have been proposed: (a) SMAD/TGF-*β* pathway, (b) WNT/*β*-catenin signaling, and (c) ECM integrin signaling cascade. In any case, these diverse EMT routes render upregulation of specific sets of transcription factors, including SNAIL, SLUG, ZEB, and TWIST, that would further control the cellular conversion process [20, 38, 39].

CSCs and non-CSC populations have also been proved able to interconvert each other depending on external stimuli, namely, factors coming from the microenvironment or in response to treatment. Some examples of differentiated cells undergoing this reversion process to become CSCs, or cells in an intermediate state showing stem-like properties, have been reported for colorectal cancer, breast cancer, and melanoma, among others [17, 40–42]. Interestingly, two of the EMT pathways (SMAD/TGF-*β* and WNT/*β*-CATENIN) have been associated also with the acquisition of stem-like properties [13].

**Figure 3:**
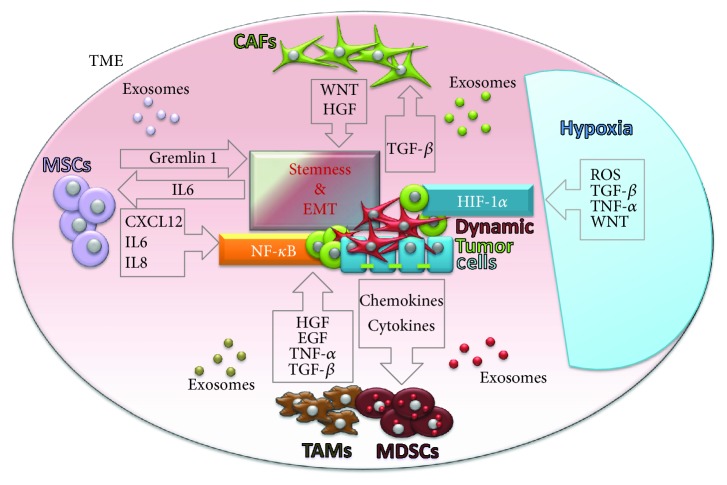
Tumor microenvironment and cancer cell phenotype. Schematic representation of TME influence on *stemness* and mesenchymal properties of cancer cells. The dynamic phenotype of cancer cells (*stemness*, EMT) is regulated by several signaling pathways. TGF-*β* and NF-*κ*B signaling pathways are activated by different microenvironmental factors like MSCs, CAFs, TAMs, MDSCs, or hypoxia. Exosomes derived from respective cell types play an important role in intercellular paracrine communication.

Moreover, another important common activator of CSC reversion and EMT is hypoxia (Figure 3). Hypoxia induces the overexpression of OCT4 that in turns triggers a molecular cascade leading to enrichment of cells with CSC-like phenotype in melanoma [43]. Hypoxic condition also induces the overexpression of hypoxia-inducible factors (HIFs), which can directly induce EMT in various cancer models, mostly conducted by the HIF-1*α* factor (Figure 3) [44, 45]. Importantly, HIF-1*α* can directly increase NOTCH signaling, enhancing *stemness* [46]. Hypoxia also promotes CSC survival and EMT through reactive oxygen species- (ROS-) activated stress response pathways and through ROS-induced TGF-*β* and TNF-*α* signaling pathways, in breast cancer (Figure 3) [47]. In glioma cells, the activation of TGF-*β* as well as WNT signaling pathways by hypoxia induces *stemness* by promoting an undifferentiated cellular state [48]. Furthermore, hypoxia seems sufficient to promote CSC phenotype and invasion and accelerate metastatic outgrowth in liver tumor cells after surgery. In addition, transcription factors recognized as pluripotency markers in embryonic stem cells such as NANOG, SOX2, and c-MYC have been reported to be upregulated in the acquirement of the CSC profile [49].

Despite some scraps of evidence from distinct tumor types showing the acquirement of *stemness* properties by differentiated cells in specific conditions, the general process by which differentiated tumor cells undergo a dedifferentiation process is still far from being completely elucidated. However, what seems clear is that the dynamism described for CSCs is analogous to the dynamism observed for EMT processes. Despite that signals and subsequent pathways triggering both processes are not necessarily shared, the acquisition of CSC phenotype and EMT partially overlaps, which goes in line with partial EMT phenotype and the CSC window theory discussed before (Figure 2). Nonetheless, the fact that signaling cascades for both processes differ by enhancing the expression of distinct subsets of transcription factors is remarkable. Therefore, although in some cases EMT and non-CSC to CSC reversion produce similar responses related with an increased malignancy of the disease, they should be considered distinct processes, both highly dependent on the cancer type.

## 3. Tumor Microenvironment and Cancer Cell Phenotype

Another important modulator of the phenotypic plasticity of cancer cells may come from the tumor microenvironment (TME), also called tumor niche (Figure 3). TME is composed of a complex network of stromal, immune, and inflammatory cells; soluble factors; signaling molecules; and the extracellular matrix [50]. Both cellular and noncellular components of the tumor niche contribute to maintaining the *stemness* of tumor cells and regulating EMT/MET and CSC plasticity [45, 51, 52].

The most abundant cell population within TME are fibroblasts [53]. Solid evidences show that cancer cells are capable of producing factors, like TGF-*β*, that once secreted to the TME can transform normal fibroblasts into cancer-associated fibroblasts (CAFs) [54]. CAFs have a battery of unique features when compared with normal fibroblasts that promote cancer progression [55]. It has been demonstrated that TGF-*β* is carried to the tumor stroma by cancer cells, enhancing CAF phenotype. Once activated, CAFs promote tumor cell progression by multiple mechanisms, in a bidirectional crosstalk between CAFs and tumor cells [56]. One of the most important players in cell-to-cell communication in the TME are exosomes [57]. Exosomes are specialized membranous nanosized vesicles (30–150 nm) derived from endocytic compartments that are released by many cell types. They contain sophisticated RNA and protein cargos from the cell of origin, enabling intercellular communication [58]. Exosomes released by activated CAFs have been associated with the promotion of EMT, *stemness*, and angiogenesis in prostate tumors [59–61]. Special relevance has been attributed to the WNT pathway, a crucial signaling cascade for these processes. The upregulation of WNT10b in CAF exosomes induces EMT of breast cancer cells [62]. A study with endometrial cancer cells has also demonstrated that upregulation of WNT10b in CAFs results in increased migration and aggressiveness of tumor cells [63]. Besides, in lung cancer models, CAFs obtained from lung cancer tissue produce hepatocyte growth factor (HGF), thereby activating the EMT-related c-Met pathway (Figure 3) [64].

Moreover, TME also contains mesenchymal stem cells (MSCs) that are considered key regulators of tumoral physiology through multiple mechanisms [65–67]. These multipotent stromal cells are implicated in the restoration of CSCs in the TME. Similar to CAFs, MSCs can promote cancer *stemness* and EMT phenotype also through TGF-*β* [68] Moreover, MSCs can stimulate tumor progression by producing Gremlin 1 to promote the undifferentiated state of cancer cells [69]. Furthermore, MSCs can provide tumor cells with CSCs properties by suppressing FOXP2 expression [70]. Exosomes released by MSC cells are important for communication of MSCs with TME, although further studies are needed to better elucidate completely their role (Figure 3). Another area of great interest is the influence of the TME in modulating tumoral immunity [68]. Accumulating data is pointing out that tumor-polarized immune cells resident in the TME enhance EMT phenotype and ultimately promote migration and invasion of CSCs [71].

The TME is characterized by chronic inflammation which leads to a phenomenon called immunosuppression in the tumor niche that stimulates tumor cell proliferation and metastasis. Tumor-associated macrophages (TAMs) and myeloid-derived suppressor cells (MDSCs) are an example of immunosuppressive cell types recruited by chemokines and cytokines that are secreted by cancer cells. TAMs are derived from polarized macrophages that acquire protumor phenotypes that enhance tumor growth and metastasis [72]. Similarly to previous examples, tumor-derived exosomes have been shown to play a key role in macrophage polarization. Within inflammatory TME, TAMs and CD4^+^ T cells secrete TNF-*α* which upregulates NF-*κ*B signaling, induce EMT, and increase the crosstalk with the TGF-*β* signaling pathway, stimulating *stemness* [71]. In agreement to this, gastric cancer-derived exosomes have been shown to induce NF-*κ*B activation in macrophages promoting the proliferation of gastric cancer cells. Similar results show that breast cancer-derived exosomes also stimulate the NF-*κ*B pathway in macrophages [73]. On the other hand, MDSCs are a heterogeneous population of cells from monocytic and granulocytic origins, which are also involved in promoting EMT and in CSC maintenance [74]. Indeed, in a spontaneous murine model of melanoma, MDSCs induce EMT via TGF-*β*, EGF, and HGF signaling [75]. Similarly, platelet-derived TGF-*β* secreted by MDSCs activates TGF-*β*/Smad and NF-*κ*B pathways in lung cancer cells, resulting in EMT and enhanced metastasis *in vivo*, in lung cancer models [76, 77].

## 4. Implication of Cancer Cell Phenotypes in Anti-Tumoral Treatment Strategies

Understanding the tight relationship among CSCs, EMT, and the tumoral microenvironment opens the door to new strategies for developing more effective anticancer treatments.

Because many CSC-related pathways are involved also in EMT, new treatments should eliminate CSCs while reverting the EMT phenotype and vice versa. For example, in order to target EMT, different strategies have been reported, usually targeting (i) adhesion-related proteins (e.g., E-cadherin), (ii) microenvironment factors (e.g., SPARC), (iii) cell membrane molecules (e.g., integrins, TGF-*β*), (iv) intracellular transcription factors (e.g., ZEB, SNAIL, SLUG, TWIST, and E47), (v) microRNAs (e.g., miRNA200, miRNA29), and a wide range of other possibilities [78]. On the other hand, the elimination of CSCs is pursued through different therapeutic strategies involving signaling pathways related with (i) CSC survival and proliferation pathways (e.g., PI3K-AKT, JAK/STAT, and NF-*κ*B) and (ii) signals linked to the *stemness* properties of CSCs, like self-renewal and pluripotency (e.g., Notch pathway, WNT pathway, and Hedgehog signaling) [5]. However, the molecular pathways studied as potential EMT targets are also involved in CSCs *stemness*, and the ones studied as CSC pathways are usually representative of EMT. Studies characterizing the effect of specific molecular players on the regulation of both CSC malignancy and EMT occurrence are still scarce. As an example, the PI3K-AKT pathway regulates the expression of TWIST, one of the most important transcription factors regulating EMT; however, the same pathway is also reported as crucial for *stemness* properties and CSCs survival [79].

As referred, CSCs and EMT cells partially share signaling pathways of EMT and *stemness* and since CSCs could undergo EMT, it is virtually impossible to characterize a therapeutic target or approach as CSC- or EMT-specific. Moreover, many studies regarding treatments directed against CSCs do not assess the therapeutic effect on EMT and vice versa, making more difficult to comprehend the interactive effects between both phenomena.

An example is Nestin, a class VI intermediate filament protein involved in mitosis. It was originally described as a neural stem cell/progenitor cell marker. However, expression of Nestin has been reported to be associated with migration and metastasis of various types of tumors and as a CSC marker [80–83]. Transfection of the tumor cancer cell line PANC-1 with a short hairpin RNA (shRNA) targeting NESTIN results in decreased NESTIN expression, increased expression of filamentous F-actin and E-cadherin, reduction of cell migration and invasion abilities, and less formation of metastasis in vivo, demonstrating its involvement in EMT [80]. Additionally, NESTIN and CSC markers like ALDH1A1 and ABCG2 are found overexpressed in metastasis-derived cancer cells presenting low levels of E-cadherin. NESTIN silencing in pancreatic cancer results in reduced sphere formation, tumor growth, and metastasis development, not only suggesting the correlation between the CSC-like phenotype and EMT but also validating NESTIN as a therapeutic target [84].

The effects of a drug in CSCs and also in the EMT process are dependent on the cell and cancer type. In triple-negative breast cancer (TNBC), salinomycin was described to cause marked suppression of cell migration and invasion as well as inhibition of mammosphere formation and effective reduction of the CD44^+^/CD24^−^ stem-like/mesenchymal subpopulation [85]. On the other hand, in case of head and neck squamous cell carcinoma, salinomycin treatment induces apoptosis and decrease in stem cell properties, despite the activation of EMT via AKT [86]. These observed differences between studies and cancer type could be explained by the previously referred *stemness* window theory (Figure 2).

The best strategy to prevent tumor remission should be the elimination of all kinds of aggressive cells within the tumor together with the bulk tumor cells since these cells have interconversion capacity and could originate new clones of CSCs or mesenchymal cell via the EMT process. Therefore, the ultimate goal for the cancer treatment field is to find the way to reach all types of cancer cells. This could be achieved by treatment protocols implying combination of various therapeutic molecules, a combination of gene therapy approaches, or the use of targeted vectors decorated with the most specific ligands found for each type of cell.

Nowadays, different therapeutic approaches have been proposed to target CSCs and/or EMT, ongoing different development stages (Table 1). Since this therapeutic approach is still in its infancy, the majority of present studies are at the preclinical phase, with a small percentage enrolling clinical evaluation. More examples of treatments under clinical trials against CSCs and/or EMT can be found at [5].

## 5. Summary

The *stemness* of CSCs, non-CSC reversion to CSCs, and EMT processes are regulated by similar signaling pathways. Provided data show that when TGF-*β* and NF-*κβ* signaling cascade is activated by different microenvironmental factors, cancer cells from various cancer types tend to undergo EMT, and this is frequently accompanied by a maintenance of a CSC stem phenotype. Based on this knowledge, strategies to prevent tumor remission should carefully consider not only eliminating potential aggressive CSCs and EMT cells within the tumor but also targeting those signaling pathways responsible for the interconversion capacity of non-CSCs to new CSCs and mesenchymal cells via EMT activation. This can be undergone through a combination of molecules, a combination of gene therapy approaches, or the use of targeted vectors decorated with the most specific ligands found for each cell type.

## Figures and Tables

**Table 1:** Different cancer therapeutic approaches based on CSCs and/or EMT.

CSC	EMT	Therapeutic target	Therapeutic approach	Cancer type	Development stage	References
	x	AKT/mTOR signaling pathway	Tunicamycin	Colon cancer	Preclinical (*in vivo*)	[87]
x	x	AKT2	siRNA	Breast cancer	Preclinical (*in vivo*)	[79]
x		ALOX5	Zileuton	Leukemia	Clinical (phase 1)	^∗^ NCT02047149 ^∗^ NCT01130688
x		Bmi-1	Nigericin	Nasopharyngeal carcinoma	Preclinical (*in vivo*)	[88]
x		Bmi-1	shRNA	Nasopharyngeal carcinoma	Preclinical (*in vivo*)	[89]
	x	EGR-1	shRNA against EGR-1	Breast cancer Lung cancer Liver cancer	Preclinical (*in vitro*)	[90]
	x	EGR-1	Oxytocin	HNSCC	Preclinical (*in vitro*)	[91]
	x	EGR-1	Syntactic catalytic DNA	Breast cancer	Preclinical (*in vivo*)	[92]
	x	EGR-1	2′-Benzoyloxycinnamaldehyde	Colon cancer	Preclinical (*in vivo*)	[93]
	x	EGR-1	siRNA	Colon cancer	Preclinical (*in vivo*)	[93]
x		Hedgehog signaling	Cyclopamine	Glioblastoma	Preclinical (*in vivo*)	[94]
	x	HMGA2	LBH589	Prostate cancer	Preclinical (*in vivo*)	[95]
x		IAP family	AT-406, SM-164, and TRAIL	Nasopharyngeal carcinoma	Preclinical (*in vivo*)	[96]
x	x	JAK 1/2	Ruxolitinib	Pancreatic cancer Ovarian cancer	Preclinical (*in vivo*)	[19, 97]
x		Krüppel-like factor 5	Metformin	TNBC	Preclinical (*in vivo*)	[98]
	x	Lysine-specific demethylase 1	Pargyline	Prostate cancer	Preclinical (*in vivo*)	[99]
x		mTOR	Rapamycin	Neuroblastoma	Preclinical (*in vivo*)	[100]
x		n.d.	Metformin	Gastric cancer Breast cancer Ovarian cancer	Preclinical (*in vivo*)	[101–103]
x		n.d.	Salinomycin	HNSCC	Preclinical (*in vitro*)	[86]
x		n.d.	Salinomycin analogs	Breast cancer Colon cancer	Preclinical (*in vitro*)	[104]
x		n.d.	EpCAM/CD3 antibody	Pancreatic cancer	Preclinical (*in vivo*)	[105]
		n.d.	Anti-CD33 antibody	Glioblastoma	Preclinical (*in vivo*)	[106]
x	x	n.d.	Quercetin	Pancreatic cancer	Preclinical (*in vivo*)	[107]
x		n.d.	All-*trans* retinoic acid	Gastric cancer	Preclinical (*in vivo*)	[108]
x		n.d.	Mithramycin	Various neoplasms	Clinical (phase 2)	^∗^ NCT02859415
x		n.d.	Drug combination	Glioblastoma	Clinical (phase 1)	^∗^ NCT02654964
	x	n.d.	Epirubicin + cisplatin + capecitabine	Gastric cancer	Clinical (phase 3)	^∗^ NCT01697072
x		NADH dehydrogenase	DECA-14	Neuroblastoma	Preclinical (*in vivo*)	[100]
x	x	Nestin	shRNA	Glioblastoma Lung carcinoma Pancreatic cancer	Preclinical (*in vivo*)	[80–82, 109]
x	x	Nestin	siRNA	Pancreatic cancer	Preclinical (*in vivo*)	[84, 110]
x	x	p53 mutant cells	Metformin	Ovarian cancer FTPPC, pancreatic cancer	Clinical (phase 2)	^∗^ NCT01579812 ^∗^ NCT02978547
x	x	PI3K-AKT; ERK1/2 pathways	LY294002; U0126	Breast cancer	Preclinical (*in vitro*)	[111]
	x	SNAIL	Trichostatin A	Lung cancer	Preclinical (*in vitro*)	[112, 113]
	x	Snail-p53 interaction	GN-25; GN-29	Pancreatic cancer Lung cancer	Preclinical (*in vivo*)	[114]
x		STAT3	LLL12; shRNA	Breast cancer	Preclinical (*in vivo*)	[115]
x		STAT3	BBI608	Various cancers	Preclinical (*in vivo*)	[116]
x	x	STAT3	Salinomycin	Breast cancer	Preclinical (*in vitro*)	[85]
x	x	STAT3 pathway	Oncostatin M	Hepatocellular carcinoma	Preclinical (*in vivo*)	[117]
x		WNT pathway	Nigericin	Lung cancer	Preclinical (*in vitro*)	[118]
x	x	ZEB1	shRNA	Pancreatic cancer	Preclinical (*in vivo*)	[19]

n.d.: not described. ^∗^ClinicalTrials.gov identifier. Abbreviations: HNSCC: head and neck squamous cell carcinoma; FTPPC: fallopian tube, primary peritoneal cancer.

## Abbreviations

TME: Tumor microenvironment
CAFs: Cancer-associated fibroblasts
MSCs: Mesenchymal stem cells
TAMs: Tumor-associated macrophages
MDSCs: Myeloid-derived suppressor cells.
